# Agricultural By-Products, Biowastes, and Other Biogenic Materials as Bio-Rejuvenators for Aged Bituminous Binders: Mechanisms, Performance, and Challenges

**DOI:** 10.3390/polym18141752

**Published:** 2026-07-17

**Authors:** Gholam Hossein Hamedi, Ozgur Ozcan, Sedat Ozcanan, Abdulgazi Gedik

**Affiliations:** 1 Department of Civil Engineering, Faculty of Engineering, University of Guilan, Rasht 41996-13776, Iran; hamedi@guilan.ac.ir; 2 Department of Civil Engineering, Faculty of Engineering, Sirnak University, 73000 Sirnak, Turkey; sozcanan@sirnak.edu.tr; 3 Department of Civil Engineering, Faculty of Engineering and Natural Sciences, Malatya Turgut Ozal University, 44900 Malatya, Turkey; abdulgazi.gedik@ozal.edu.tr; 4 Department of Transportation Engineering, Faculty of Civil Engineering, Istanbul Technical University, 34469 Istanbul, Turkey

**Keywords:** asphalt binder, rejuvenation, aged bitumen, recycling, biowaste

## Abstract

Asphalt binders become stiffer and more brittle during aging, increasing their susceptibility to fatigue and thermal cracking. Rejuvenation is therefore a key strategy for restoring aged bitumen and enabling the effective use of reclaimed asphalt materials. This review examines agricultural by-products, biowastes, and other biogenic materials as bio-rejuvenators for aged bituminous binders. The reviewed materials are classified according to their source, processing route, functional role, chemical characteristics, rejuvenation mechanism, performance effects, and practical limitations. The main groups include waste cooking oils, virgin vegetable oils, reactive bio-oils, biomass-derived bio-oils, agricultural and forestry residues, tree-resin-derived products, animal-based rejuvenators, and other organic waste-derived materials. The literature indicates that these materials can restore aged bitumen through light fraction replenishment, colloidal rebalancing, diffusion, asphaltene deagglomeration, chemical interaction, and anti-aging effects. Waste cooking oils and agricultural/forestry residue-derived rejuvenators provide particularly strong recovery of conventional binder properties, whereas reactive bio-oils and tree-resin-derived systems offer a more balanced rheological response and cracking–rutting balance. However, their effectiveness strongly depends on dosage, feedstock variability, binder compatibility, processing route, and aging condition. Overall, bio-rejuvenators offer a promising pathway for sustainable asphalt recycling, but their practical implementation requires standardized dosage selection methods, long-term aging assessment, mixture- and field-scale validation, and life-cycle evaluation.

## 1. Introduction

Asphalt mixtures constitute one of the most extensive and indispensable parts of roads, where performance and longevity are predominantly determined by the bituminous binder. Bitumen is a fossil-based material derived from crude oil and is therefore nonrenewable, making it vulnerable to long-term resource depletion and price fluctuations. Global crude oil demand increased by nearly 1.5 million barrels per day in 2024 and reached an average of 103.7 million barrels per day. In parallel, global bitumen production reached approximately 174 million metric tons in 2025 [1,2]. Coupled with the growing global demand for petroleum products and the impending depletion of crude oil reserves, the supply of high-quality bitumen is becoming increasingly constrained. These challenges have compelled the development of emerging technologies and strategies that both prolong the in-service life of asphalt pavements and promote the reuse of discarded and end-of-life asphalt mixtures.

**Figure 1:**
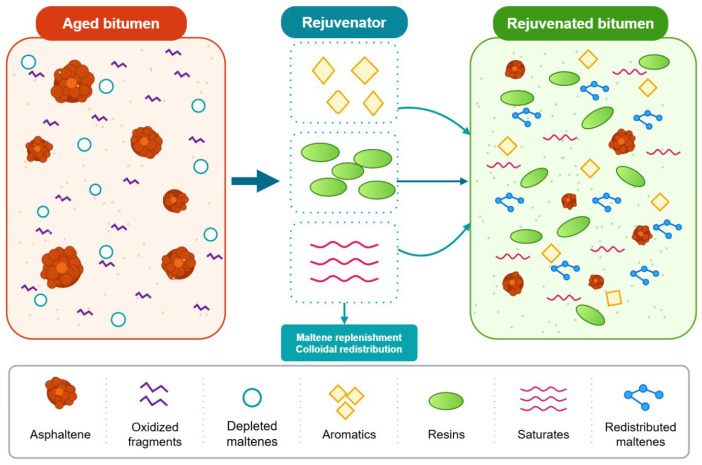
A schematic representation of the bitumen rejuvenation mechanism (this figure was drawn by the authors using draw.io (https://app.diagrams.net (accessed on 9 July 2026)) and adapted from [8]).

In pursuit of sustainable pavements, bitumen rejuvenation has recently emerged as a technically effective and economically attractive approach for rehabilitating the functional properties of aged bitumen, particularly in reclaimed asphalt pavement (RAP) and reclaimed asphalt shingle (RAS) applications. Bitumen rejuvenation involves the replenishment of maltene fractions lost during thermo-oxidative aging. This process reduces binder consistency and helps recover ductility, adhesion, cohesion, and cracking resistance [3]. Unlike simple softening agents, rejuvenators aim to rebalance the chemical and colloidal structure of aged bitumen. Aging increases asphaltene aggregation and oxidation products while reducing the effective role of the maltene phase. Maltene-rich rejuvenators can therefore compensate for lost light components and promote colloidal redistribution [4,5]. As a result, the aged binder may partially regain a more dispersed and balanced structure. The general working mechanism of bitumen rejuvenation is shown in Figure 1. In this context, rejuvenation plays a central role in enabling high-RAP-content mixtures, reducing dependence on fresh binder, and supporting circular material flows within the asphalt industry [6]. Furthermore, it offers significant life-cycle benefits from an environmental perspective. Rejuvenation not only decreases the landfill disposal burden of scrapped asphalt mixtures but also reduces greenhouse gas emissions and energy consumption [7]. This approach aligns with the principles of the circular economy and sustainable infrastructure, where waste minimization and resource efficiency are prioritized. Last but not least, rejuvenation contributes to lower mixing and compaction temperatures, enhanced workability, and extended pavement life, which in turn lead to reduced maintenance frequency and a lower overall environmental footprint of road networks.

In recent years, research has shifted from petroleum-based rejuvenators toward bio-based alternatives derived from agricultural by-products and biowastes. This shift is driven by environmental regulations, sustainability targets, and the rising cost of fossil-derived materials [9]. Agricultural residues, waste cooking oils, lignocellulosic biomass, and forestry by-products are attractive because they are renewable, relatively low-toxic, and compatible with circular economy principles [10]. Many of these materials can restore aged binder properties while valorizing organic wastes that would otherwise be landfilled or incinerated. Some also contain antioxidant compounds and functional groups that may improve the aging resistance of rejuvenated binders [11,12]. Another standout advantage is their ability to achieve the desired rejuvenation effect at relatively low dosages [13,14].

**Figure 2:**
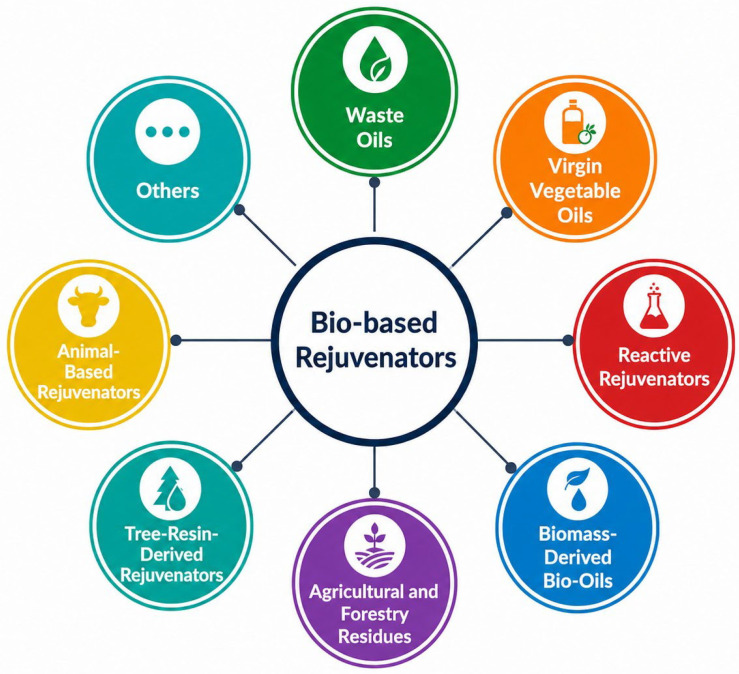
Bio-rejuvenators for aged asphalt binders (this figure was drawn by the authors using Microsoft^®^ PowerPoint^®^ for Microsoft 365 MSO).

The incorporation of agricultural by-products and biowastes into aged binders represents a strategic pathway toward more resilient, cost-effective, and environmentally responsible pavement technologies [15]. A thorough understanding of the mechanisms, benefits, and limitations of these emerging bio-rejuvenators is therefore essential for advancing sustainable pavement engineering and for safeguarding the long-term viability of asphalt infrastructure in a resource-constrained future [16]. Accordingly, this paper presents a state-of-the-art review that categorizes and elucidates the most prominent bio-rejuvenators (Figure 2).

Several recent review studies have examined bio-based materials for asphalt rejuvenation from different perspectives. Some of these reviews focused on bio-rejuvenators in asphalt pavements, waste asphalt rejuvenation, high-RAP mixtures, and general rejuvenator classifications by considering dosage, binder- and mixture-level performance, micro- and macro-properties, and implementation challenges [10,15,17,18,19]. Other studies specifically discussed biological rejuvenation mechanisms, bio-oil composition, diffusion, functionalization, component regulation, and the interaction of plant-, animal-, algal-, and waste-derived rejuvenators with aged asphalt [7,16]. In addition, vegetable oils, waste cooking oils, and biomass-derived bio-oils have been reviewed in terms of production or upgrading processes, chemical structure, compatibility, modification/rejuvenation potential, environmental benefits, and life-cycle-related considerations [20,21,22,23].

Although these studies provide useful information on bio-rejuvenation technologies, most of them focus on either general bio-rejuvenator classes; specific material groups such as waste cooking oils, vegetable oils, or bio-oils; or performance-based evaluations of RAP mixtures. The present review differs from these studies by considering agricultural by-products, biowastes, and other biogenic materials within a unified and comparative classification framework. Rather than evaluating bio-rejuvenators only as softening agents or separate material types, this review relates their source, processing route, functional role, chemical characteristics, rejuvenation mechanism, performance effects, and practical limitations. In this regard, it provides a broader evaluation of how different bio-rejuvenator groups restore aged bitumen through maltene replenishment, colloidal rebalancing, diffusion, asphaltene deagglomeration, chemical interaction, and anti-aging effects. It also points out unresolved issues related to dosage optimization, feedstock variability, binder compatibility, secondary aging resistance, long-term durability, field-scale validation, and standardization, which remain important for the practical use of agricultural waste-derived bio-rejuvenators in sustainable pavement recycling.

**Figure 3:**
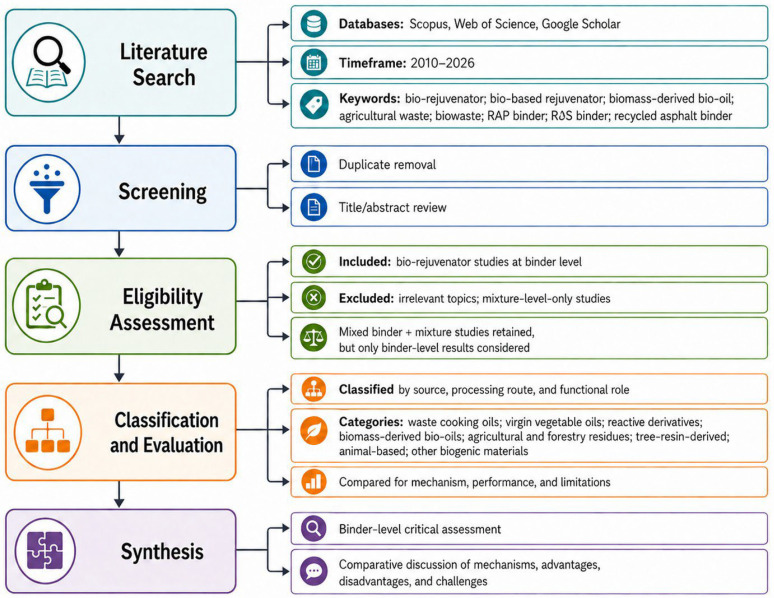
The literature search, screening, classification, and synthesis workflow used in this review (this figure was drawn by the authors using Microsoft^®^ PowerPoint^®^ for Microsoft 365 MSO).

This review was carried out as a structured and classification-based critical review focusing mainly on binder-level bio-rejuvenation studies, rather than as a fully systematic review bibliometric analysis. The literature search and selection procedure is presented in Figure 3. Studies published between 2010 and 2026 were searched using Scopus, Web of Science, and Google Scholar. The search terms included combinations of “bio-rejuvenator”, “bio-based rejuvenator”, “biomass-derived bio-oil”, “agricultural waste”, “biowaste”, “RAP binder”, “RAS binder”, and “recycled asphalt binder”.

**Figure 4:**
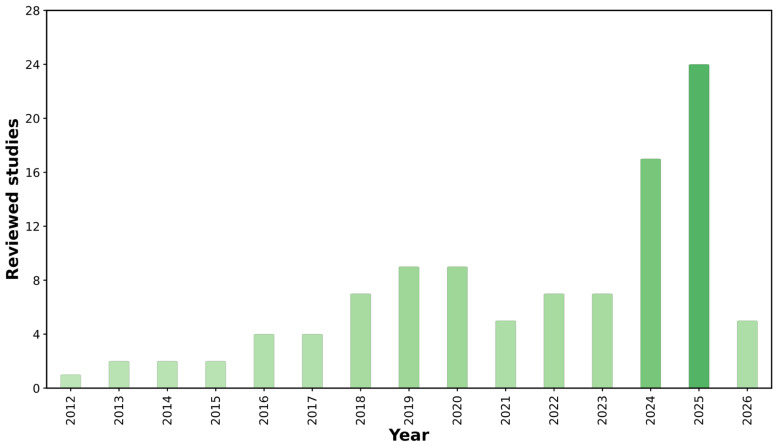
The yearly distribution of the reviewed bio-rejuvenation studies; darker green shades indicate a higher number of studies. (this figure was drawn by the authors using Microsoft^®^ Excel^®^ for Microsoft 365 MSO).

Studies were included when they investigated bio-based, waste-derived, or biogenic materials as rejuvenators for bituminous, RAP, or RAS binders. They also had to provide binder-level evidence based on rheological, chemical, thermal, microstructural, or mechanistic recovery. Studies were excluded if they focused only on mixture-level performance, used the material only as a conventional modifier, addressed biofuel or bio-oil production without asphalt application, or lacked sufficient information on rejuvenator source or composition. When both binder- and mixture-level results were presented in the same study, only the binder-level findings were used in the classification and comparative assessment. Review papers were used only for background information and contextual comparison, not as primary evidence in the classification tables. The selected studies were classified according to rejuvenator source, processing route, and functional role. They were then compared in terms of rejuvenation mechanisms, performance improvements, limitations, and practical challenges. The yearly distribution of the reviewed studies is shown in Figure 4, indicating a clear increase in binder-level bio-rejuvenation research in recent years, particularly after 2020.

## 2. Bio-Rejuvenators

### 2.1. Waste Cooking Oils

Waste cooking oils (WCOs) or post-consumer cooking oils (PCOs) have been widely investigated as sustainable bio-rejuvenators for aged asphalt binders (Table 1), particularly as alternatives to waste engine oils (WEOs). Their rejuvenating efficiency is strongly dosage-dependent. Zargar et al. reported that 3–4 wt.% WCO was sufficient to restore 40/50 aged bitumen close to the original 80/100 binder condition [24]. Similarly, 1% waste vegetable oil restored aged 20/30 asphalt to approximately 40/50 penetration grade, whereas 3% WEO was required for a comparable effect [25]. El-Shorbagy et al. found optimum dosage ranges of 3.5–4.0% for WCO and 5.5–6.0% for WEO, with the rejuvenated binders reaching PG 64–28 and showing low-temperature performance comparable to the control binder [26]. Other studies also confirmed that WCO-based rejuvenators can restore penetration, viscosity and rheological response, although the optimum dosage may increase substantially depending on aging severity, with Xinxin et al. reporting 13.4 wt.% as the optimum W-oil content for PAV-aged asphalt [27].

The rejuvenation mechanism of WCO is mainly related to diffusion, maltene replenishment, and colloidal rebalancing rather than a direct chemical reaction. WCO-derived oils contain light fractions such as saturates, aromatics, and resins, which compensate for components lost during aging and reduce the relative dominance of asphaltenes [28]. As WCO dosage increases, binders generally become softer, less viscous, and more flexible at low temperatures [27,28,39]. However, excessive softening may reduce rutting resistance; therefore, dosage control is essential [36,42].

Recent studies have provided more quantitative dosage guidance for binders containing high RAP contents. Jain and Chandrappa showed that 5.0% WCO was suitable for 50% RAP binder, while 7.5% WCO was required for 60% RAP binder, with both combinations showing rheological behavior close to that of the virgin binder [35]. In a later study, the optimum WCO ranges were reported to lie between 7.5 and 10% for 75% and 60% RAP binders and between 5 and 7.5% for 45% RAP binder [36]. Rai et al. also showed that aging severity strongly affects dosage demand: 4% waste soybean oil was sufficient for short-term aged bitumen, whereas 8–10% was required for long-term aged bitumen [32]. These results indicate that WCO dosage should be selected according to RAP content, aging level and target rheological parameters rather than penetration or softening point alone.

The chemical quality and processing route of WCO are also critical. High free fatty acid content or acid value may impair high-temperature stability, moisture resistance and long-term durability. Therefore, chemical pretreatments such as transesterification and catalytic esterification have been used to improve WCO performance. Azahar et al. reduced the acid value of WCO from 1.65 to 0.54 mL/g through transesterification, which increased the DSR failure temperature from 64 °C to 70 °C [33]. Oldham et al. similarly showed that transesterification reduced the acid value from 12 to 0.27 mg KOH/g and greatly improved moisture resistance [34]. More recently, Ye et al. reported that 6% catalytically esterified WCO increased fatigue life at 5% strain by 71.63% compared with virgin asphalt, while the creep rate at −24 °C was 4.15% higher than that for virgin asphalt and 17.84% higher than that for original WCO-rejuvenated asphalt [41].

Besides direct blending, WCO has been incorporated into microcapsules for in situ rejuvenation. In these systems, polymeric shells protect the oil during mixing and allow for gradual diffusion into the binder [29,30,31]. This delayed action can replenish depleted oily fractions over time and may provide an anti-aging benefit. Nevertheless, over-softening, rutting susceptibility, moisture sensitivity, and secondary aging should still be assessed using combined physical, rheological, chemical, and durability-related indicators [33,34,37,38,41].

### 2.2. Virgin Vegetable Oils

**Figure 5:**
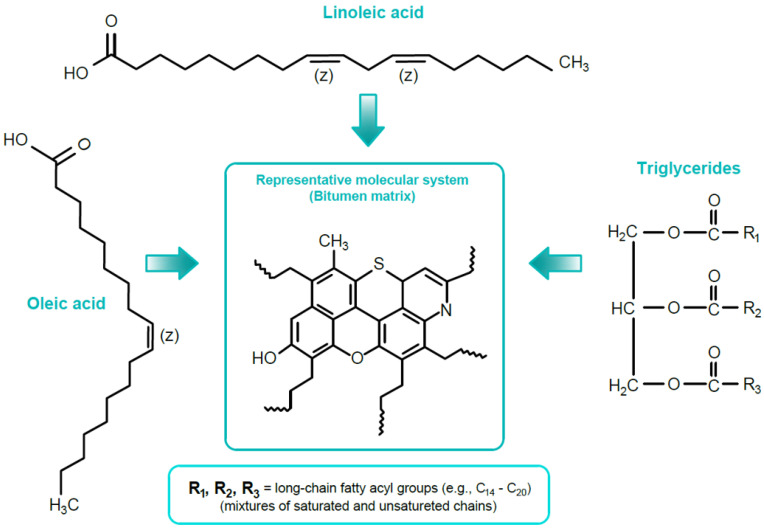
The representative chemical structure of VVO-based rejuvenators (this figure was drawn by the authors using Autodesk AutoCAD 2024 and adapted from [43]).

A variety of virgin vegetable oils (VVOs) have been used to rejuvenate aged bitumen, as they restore maltenes, enhance flexibility and workability, demonstrate chemical compatibility, and provide environmental and sustainability benefits. VVO-based rejuvenators generally consist of triglycerides and long-chain unsaturated fatty acids, mainly oleic and linoleic acids (Figure 5). These components may behave as light oil-like fractions in aged bitumen, reducing stiffness and improving molecular mobility [43,44].

When VVO-based rejuvenators are combined with trimethylolpropane triglycidyl ether, they become more effective in recovering the overall high- and low-temperature performance of polymer-modified bitumens [45]. Babassu oil at 3–7% effectively rejuvenated recovered RAP binders by reducing their PG from 76 °C to 64 °C for RA1 and to 52–58 °C for RA2 while lowering the Glover–Rowe parameter by about 90.5–91.9% and 90.5–94.7%, respectively. This softening and crack mitigation effect enabled high-RAP mixtures containing 56–70% RAP, with fatigue resistance improved at strain amplitudes above 5% and moisture/Cantabro abrasion responses remaining statistically comparable to the control mixture [46]. Similarly, date seed oil, containing more than 60% fatty acids, improved the performance of RAP-blended binders by reducing stiffness and enhancing fatigue and low-temperature response. The most favorable results were reported for 20–30% RAP binder blends, where fatigue life increased by up to 15%, while 40% RAP mixtures required further optimization due to rutting and performance limitations [47,48]. Elkashef et al. [49] showed that 6 wt.% soybean oil-derived rejuvenators reduced the binder grade from PG 58–28 to PG 46–34 and shifted the critical low-temperature grade from −29.9 °C to −37.6 to −39.4 °C, consistent with a lower glass transition temperature and improved low-temperature cracking resistance. Zhou et al. [50] quantitatively evaluated soybean and palm oil-based rejuvenators in terms of wetting, diffusion, and thermodynamic compatibility. Palm oil exhibited the most negative free energy of mixing (−889.8 J/mol), followed by soybean oil I (−730.4 J/mol), indicating stronger thermodynamic compatibility and greater potential for restoring the molecular and colloidal structure of aged asphalt binder. Specifically, soybean oil possessed solubility parameters closely matching those of aged asphalt binder, leading to superior compatibility and high solubility [43]. In addition, corn and soybean oil-based rejuvenators were reported to reduce viscosity and stiffness more effectively than mineral oil-based rejuvenators. These oils also improved fatigue and low-temperature cracking resistance, while a dosage range of 6–8% was generally suggested when high-temperature rutting resistance was also taken into account [51,52].

Palm oil-based materials and their by-products have also been evaluated as rejuvenating agents. Reference [53] showed that 3.0% crude palm oil, soft stearin, and hard stearin reduced the stiffness of binder blends containing 70% virgin binder and 30% aged binder while providing Jnr values comparable to or lower than those of the virgin binder and higher elastic recovery after rolling thin film oven (RTFO) aging. However, their effectiveness decreased after long-term aging, with crude palm oil showing the most favorable cracking resistance among the palm-based products. Non-edible oils have also been used successfully; Nayak and Sahoo [54] reported that approximately 5% pongamia oil or composite castor oil restored aged binder performance, with pongamia oil being more effective for fatigue resistance and composite castor oil showing better rutting resistance and thermal stability above 200 °C. In general, vegetable oil rejuvenators have been associated with lower weight loss, a reduced complex modulus, a limited increase in stiffness after aging, and better resistance to short-term aging compared with mineral oil rejuvenators [53,55]. However, their effectiveness is strongly dependent on rejuvenator type, dosage, aging level, and incorporation method. Therefore, long-term performance retention and the selection of a suitable addition method should also be considered to obtain the expected rejuvenating effect [56].

Although vegetable oil-based rejuvenators can recover aged binder properties, their dosage should be carefully controlled. Excessive use may reduce high-temperature stiffness and rutting resistance; therefore, binder recovery and permanent deformation resistance should be considered together [51,52]. Their effectiveness may also decrease after long-term aging and vary with oil type, chemical composition, and aging level [53,56]. Moreover, oxygenated functional groups in some vegetable oils may increase aging susceptibility in certain binder systems [53].

### 2.3. Reactive Rejuvenators

Chemically modified bio-oils can be regarded as reactive or semi-reactive bio-rejuvenators. This group includes epoxidized vegetable oils, partially epoxidized oils, acrylated phenolic bio-oils, compound bio-oil formulations, and bio-oil-assisted reactive systems. Their effect is not limited to physical softening. Depending on their functional groups, they may improve diffusion, interact with oxidized structures, reduce asphaltene aggregation, and restore molecular mobility. In this context, chemical and molecular indicators such as the sulfoxide index, molecular weight distribution, large molecular size fraction, and polydispersity have been reported to correlate with the rheological recovery of bio-rejuvenated binders [57].

Acrylated and non-soybean-based bio-oils provide important alternatives to ESO-based rejuvenation. Uchoa et al. evaluated epoxidized castor oil (ECO) and acrylated cashew nut shell liquid (ACNSL) and reported that both bio-rejuvenators reduced aging-related sulfoxide groups in aged binders. Based on multiple stress creep and recovery (MSCR) results, 7% ACNSL provided the best rutting resistance, while 10% ACNSL gave the highest fatigue-related response, indicating that moderate dosages of approximately 7–10% may provide a better balance between stability and elasticity [58]. Compound bio-oil systems have also shown promising results. In a castor oil vegetable asphalt (COA)/ESO system, 8% total bio-rejuvenator was used, and the optimum ESO/COA ratio was 75/25 for base asphalt and 50/50 for styrene–butadiene–styrene (SBS)-modified asphalt. For the base asphalt system, penetration increased by 7.9 dmm, and the softening point increased by 4.6 °C, showing that bio-oil-based rejuvenation can restore consistency without necessarily causing a severe loss in high-temperature stability [59].

**Figure 6:**
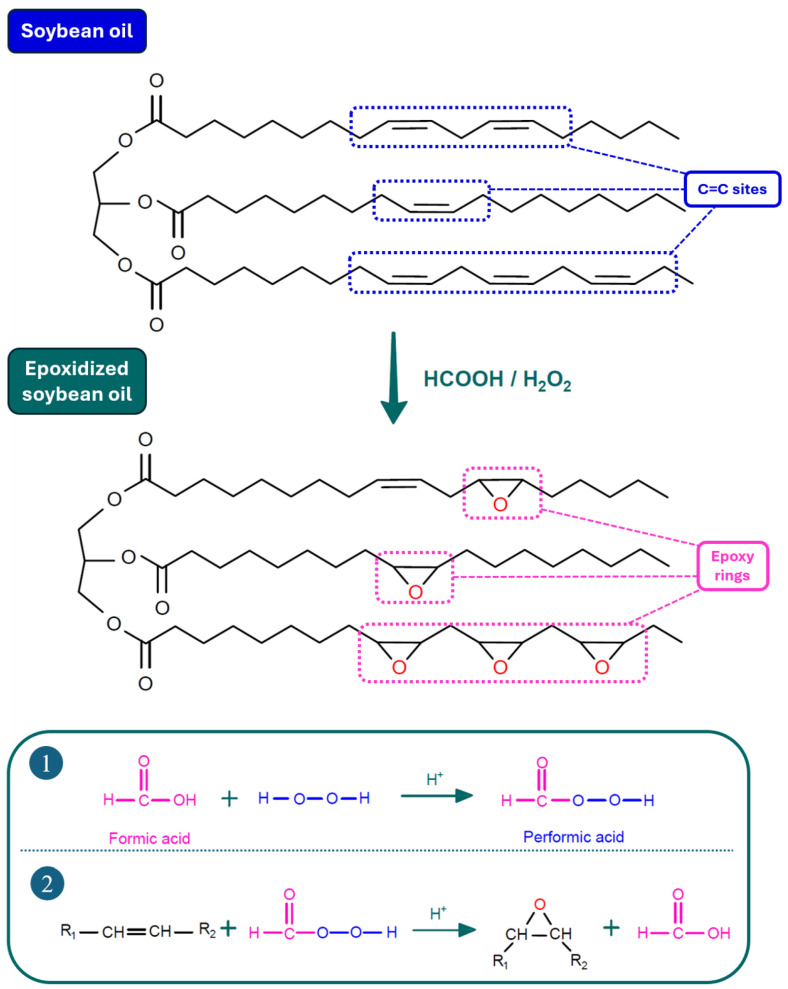
The epoxidation pathway of soybean oil and formation of oxirane-functionalized epoxidized soybean oil (this figure was drawn by the authors using Autodesk AutoCAD 2024 and Microsoft PowerPoint and adapted from [65]).

Epoxidation is a functionalization process in which the carbon–carbon double bonds of vegetable oils are converted into reactive epoxy groups (Figure 6). ESO derivatives still represent the most extensively studied subgroup, but their performance is strongly controlled by epoxy/oxirane value and dosage. ESO used at 1–7 wt.% improved the diffusion of rejuvenating components into aged bitumen, and the mixture containing 7 wt.% ESO approached the fatigue, cracking, and water stability performance of the virgin hot-mix asphalt; however, the ESO content was recommended not to exceed 7 wt.% because of the risk of reduced rutting resistance [60]. Sub-epoxidized soybean oil (SESO) was evaluated at 6% and 12% dosages, and 6% SESO with a high epoxide value was reported as the most balanced formulation. Nevertheless, 12% low-epoxide SESO produced the highest fatigue life improvement, increasing fatigue life by 119.2%, 126.9%, 316.5%, and 341.9% at strain levels of 2.5%, 5%, 7.5%, and 10%, respectively [61]. Partially epoxidized soybean oil (PESO) also showed an epoxy value-dependent response; PESO-L reduced the sulfoxide index by 38.6%, while PESO-H decreased the long-term aging index by 38.5% after pressure aging vessel (PAV) aging [62]. At the micro-mechanical level, increasing epoxy functionality reduced the Derjaguin–Muller–Toporov (DMT) modulus of aged asphalt by 24.4%, increased adhesion force by 20.9%, and reduced the sulfoxide index by more than 38%, confirming that epoxy groups contribute not only to softening but also to chemical interaction with oxidized asphalt components [63]. Field-oriented evidence also supports the practical relevance of oxirane-functionalized bio-rejuvenators: epoxidized methyl soyate and SESO enabled asphalt mixtures with 45 wt.% recycled content and approximately 30% reductions in cost, energy, and emissions; in a field project, a 40% RAP section achieved PG 64–28 compared with a 25% RAP control section, and the results suggested that RAP content could potentially be increased to 60% with acceptable performance [64].

For aged SBS-modified binders, bio-oil-based rejuvenation alone may be insufficient to restore the complete performance balance, especially when both bitumen aging and polymer degradation occur. ESO-containing systems have therefore been investigated to improve low-temperature performance, fatigue resistance, elastic recovery, thermal stability, and anti-aging behavior. In one study, ESO-assisted rejuvenation reduced the low-temperature relaxation modulus of aged SBS-modified asphalt by 35.8%, but ESO alone could not fully restore all performance parameters, indicating the need for optimized bio-oil dosage and formulation design [66]. Similarly, ESO combined with polymer-assisted rejuvenation improved rutting resistance, elastic recovery, fatigue resistance, and thermal stability, while Fourier transform infrared spectroscopy (FTIR), atomic force microscopy (AFM), and thermogravimetric analysis (TGA) results confirmed improvements in chemical structure, micro-morphology, and thermal behavior [67]. Recent studies further showed that bio-oil-containing reactive systems can reduce volatile organic compound (VOC) emissions and improve rejuvenation effectiveness when their formulation is optimized, although their practical use still requires the careful control of dosage, epoxy value, reaction pathway, compatibility, processing sensitivity, cost, secondary aging resistance, and long-term field durability [68,69].

### 2.4. Biomass-Derived Bio-Oils

**Figure 7:**
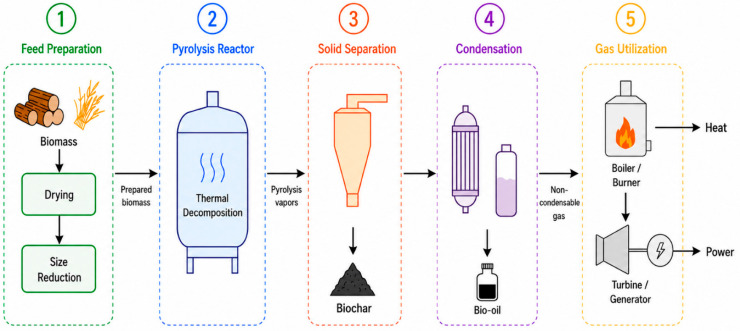
A schematic representation of the biomass fast pyrolysis process (this figure was drawn by the authors using Autodesk AutoCAD 2024 and Microsoft PowerPoint and adapted from [84]).

Biomass-derived bio-oils obtained from waste wood, sawdust, corncob, birch bark, rice husk, sugarcane bagasse, rice straw, biodiesel by-products, and other agricultural or forestry residues have been examined as rejuvenating agents for aged bitumen, as summarized in Table 2. These oils are generally produced through fast pyrolysis (Figure 7) or thermochemical liquefaction, since these processes convert biomass wastes into low-viscosity products containing light fractions, phenolic compounds, aromatics, and oxygenated components. Such constituents can help compensate for the compositional imbalance of aged binders.

Fast pyrolysis is one of the most common routes for producing biomass-derived bio-oils, in which lignocellulosic wastes are rapidly thermally decomposed under oxygen-limited conditions to obtain low-viscosity oils rich in light compounds, aromatics, oxygenated species, and phenolic constituents. Waste wood oil (WWO) and sawdust-derived bio-oil have been among the most frequently investigated pyrolysis products. For PAV-aged binders, waste wood bio-oil at 10, 15, and 20 wt.% reduced viscosity and increased the viscous component of aged asphalt; at 150 °C and 165 °C, the 15 wt.% dosage reduced viscosity by 41.0% and 59.7% compared with the virgin binder, while 15 wt.% was recommended as an effective dosage because further increases produced limited additional benefit [72]. Similarly, sawdust-derived bio-oil at 10, 15, and 20 wt.% decreased viscosity and activation energy, while the rutting index of PAV-aged PG 58–28 and PG 64–22 binders decreased by 75.5% and 77.2% on average between 52 and 76 °C, respectively; 15 wt.% and 20 wt.% were recommended for PG 58–28 and PG 64–22 aged binders [72]. Other pyrolysis-derived oils also showed feedstock-dependent responses. Corncob and birch bark oils were generally effective at 5–10 wt.%, with 10 wt.% birch bark oil giving the strongest recovery in viscosity, activation energy, stiffness, and low-temperature parameters [73]. For sugarcane bagasse oil (SBO) and rice straw oil (RSO), approximately 15 wt.% SBO and 10–15 wt.% RSO produced 2–5 times higher fatigue life than aged binder at moderate to high strain levels, although this benefit was largely reduced after secondary aging [81]. Biomass-derived phenolic oil (BDPO) showed aging condition-dependent optimum dosages of 1.5 wt.% for RTFO-aged, 3 wt.% for PAV-aged, and 8 wt.% for UV-aged binders, restoring rutting, fatigue, and low-temperature cracking resistance close to unaged asphalt [83]. Comparative studies also confirmed that optimum dosages vary by oil type, with recommended ranges of 6.21–6.75% for castor oil, 5.84–6.05% for soybean oil, and 6.31–6.55% for straw oil [77,82].

Direct blending is a simpler application route in which prepared bio-oil is incorporated into aged or RAP binders without additional chemical upgrading. In this group, crop-derived bio-oils, biodiesel by-product oils, bio heating oil (BHO), and comparative biomass-based oils generally reduced stiffness and viscosity while improving fatigue, healing, and low-temperature response. For example, 1.8 wt.% crop-derived bio-oil in a 50% RAP system reduced viscosity and restored temperature performance close to virgin asphalt, while the rejuvenated mixture achieved retained strength index values of 90.8% and 89.4% after 24 h and 48 h immersion, respectively [76]. Biodiesel-derived bio-oil used up to 3 wt.% increased penetration by about 30% per 1 wt.% addition and decreased the softening point by about 2 °C per 1 wt.% addition; however, healing after a 15 min rest period was limited to 26.9% [78]. BHO at 10 wt.% enabled mixtures with 60% RAP in hot-mix asphalt and 80% RAP in warm-mix asphalt to satisfy medium/low-traffic performance requirements even after long-term aging [79].

Thermochemical liquefaction produces liquid bio-oils through the combined effect of solvents, catalysts, and controlled heating. These products are often rich in phenolic and functionalized compounds, which may improve antioxidant activity and colloidal regulation. At the molecular scale, 10 wt.% WWO disrupted oxidized asphaltene nanoclusters mainly through intercalation, whereas SLRO showed an unfavorable compression-type effect [70]. In a wood-based liquefaction system, liquefaction products, functional products, and dioctyl phthalate were optimized at a 55:10:35 ratio, and 10 wt.% wood-based rejuvenator most effectively restored penetration, ductility, viscosity, complex modulus, phase angle, rutting factor, and zero-shear viscosity [75]. Phenol-rich bio-oils produced from corncob, birch bark, sawdust, pine bark, and peanut shells at 10 wt.% also improved secondary-aged bitumen by increasing the phase angle, decreasing the complex modulus, and reducing the carbonyl index, molecular weight, and polydispersity, with their antioxidant effect being positively related to phenolic compound content [80]. Overall, biomass-derived bio-oils can provide measurable recovery in viscosity, stiffness, fatigue life, low-temperature parameters, oxidation-related indices, and molecular distribution; however, their effectiveness remains strongly controlled by production route, feedstock chemistry, dosage, aging level, and secondary aging resistance.

### 2.5. Agricultural and Forestry Residues

Agricultural and forestry residues have been evaluated as renewable sources for producing bio-rejuvenators through solvent liquefaction, pyrolysis, esterification, and functionalized bio-oil design. Cao et al. [85] showed that cashew shell oil, used as an agricultural residue-derived rejuvenator, provided the highest rejuvenation effect among the tested agents for aged SBS-modified bitumen and showed good resistance to long-term aging. This was supported by its higher initial decomposition temperature of 215 °C and lower aging indices after TFOT and PAV aging, indicating better thermal oxidative stability [85]. Yue et al. [86] reported that liquefaction products derived from poplar sawdust, wheat straw, soybean straw, Mongolian scotch pine sawdust, corn straw, and rice husk could be upgraded into rejuvenating components, with soybean straw showing the best performance under optimized liquefaction conditions. Purified and esterified liquefaction products were able to restore the penetration, stiffness, low-temperature rheological parameters, fatigue behavior, microstructure, and chemical characteristics of aged asphalt to values close to those of the virgin binder; for example, esterified liquefaction product (ELP)-10% increased penetration by approximately 118.8% compared with aged asphalt, while esterification improved ductility by about 280.0% compared with untreated liquefaction products at the same dosage [86,87]. In soybean straw-derived systems, functionalization converted alcohol-rich liquefaction products into ester-rich and less polar compounds, improving diffusion ability, ductility, relaxation capacity, and viscoelastic recovery. The recommended esterified liquefaction product content was generally reported as 9–11%, and 10% dosage restored high- and low-temperature rheological properties close to those of virgin asphalt [87,88]. Similar improvements have been reported for forest- and plant-derived systems. Pine tree derivatives restored physical and rheological properties after aging and improved high- and low-temperature performance, as well as fatigue resistance at intermediate temperatures; optimum dosages of 12.4% and 7.5% were reported for two recovered binders [89]. Cardanol- and distilled tall oil-based systems combined softening and asphaltene deagglomeration effects, resulting in improved cracking resistance, reduced molecular weight, and a more uniform asphalt microstructure than that of single-component oils. In this system, a 7:3 distilled tall oil/cardanol ratio was selected as the most suitable design, and cardanol addition increased penetration and ductility by up to 20.3% and 35.9%, respectively [90]. In addition, agricultural waste-derived biochars, such as lemon peel char and olive kernel-derived biochar, contributed to oxidative resistance, binder stabilization, high-temperature stability, elastic recovery, and moisture-related durability, depending on their use form and feedstock source. Lemon peel-derived char showed the strongest antioxidant effect, while a 6% waste edible oil methyl ester + 3% olive kernel-derived biochar system reduced accumulated strain by about 50% and retained more than 80% tensile strength after three freeze–thaw cycles [91,92]. Commercial bio-rejuvenators obtained from seed oil, cashew nut shell oil, and tall oil also improved the low-temperature cracking resistance and fracture toughness of RAP binders, although this mechanical recovery did not always indicate a true reversal of oxidation at the functional group level. At 5 wt.% dosage, these rejuvenators restored RAP binder penetration to the 50/70 target range, increased the work of fracture by up to 40–50%, and reduced fracture toughness temperature by 9–14 °C, depending on the rejuvenator type [93].

However, these rejuvenators should not be evaluated without considering their limitations. Solvent liquefaction products may contain polar groups, residues, moisture, or hydrophilic components, which can restrict their direct use and require purification, esterification, or functionalization [86,88]. Their ability to restore ductility, temperature susceptibility, and low-temperature properties may also remain limited unless their chemical composition is upgraded [86,87]. Moreover, although pine, seed, cashew, and tall oil-based rejuvenators can improve rheological or mechanical properties, aging may reduce their initial benefits, and FTIR results show that oxidation-related chemical changes are not necessarily reversed [89,93]. Biochar-based additives may also improve oxidative stability and moisture resistance, but their contribution is feedstock-dependent and may be limited in terms of direct rejuvenation or low-temperature flexibility [91,92].

### 2.6. Tree Resin-Derived Rejuvenators

As summarized in Table 3, binder-level studies on tree resin-derived bio-rejuvenators mainly focus on tall oil-based products, including tall oil fatty acids, distilled tall oil, rosin, tall oil-based commercial agents, and Sylvaroad-type products. At the binder scale, tall oil-derived fractions have been reported to restore aged asphalt mainly through softening, diffusion, and colloidal rebalancing. In particular, tall oil fatty acids showed high diffusion ability and restored rheological properties, improving low-temperature and fatigue-related responses. In contrast, rosin was more effective in improving high-temperature rutting resistance but less effective in restoring low-temperature properties [94]. Similar binder-scale findings were reported for tall oil-based rejuvenators and Sylvaroad RP1000, where these agents reduced aged binder stiffness, recovered performance grade, and improved fatigue-related rheological indicators [44,95].

Recent molecular and microstructural studies further indicate that tall oil may contribute to rejuvenation not only by physical softening. Compared with conventional aromatic oil, tall oil showed higher molecular polarity and better affinity with asphaltene aggregates. This may promote asphaltene deagglomeration by weakening π–π stacking and increasing intermolecular spacing [96]. Accordingly, tall oil-based rejuvenation can be associated with improved diffusion, compatibility, and partial recovery of the aged binder microstructure. Earlier binder-level and extracted binder studies also showed that tall oil-based products reduced the elastic modulus and maintained part of their stiffness reduction effect after secondary aging. However, the degree of recovery varied depending on rejuvenator type and aging severity [101]. More recent tall oil diffusion studies also reported that a dosage of about 5.5% could nearly restore ductility to the virgin asphalt level, while a Sylvaroad dosage study prepared 0, 3, 5, 7, and 10% rejuvenator contents and estimated 10.6% of aged binder mass as the content needed to recover the high-temperature PG of a 50/70 reference binder [94,95,96].

Nevertheless, several limitations should be considered. The performance of tree resin-derived rejuvenators depends on fraction type, dosage, diffusion ability, and compatibility with the aged binder. For example, fatty acid-rich products may improve low-temperature and fatigue-related properties, but they may reduce high-temperature rutting resistance. Rosin-rich products may show the opposite tendency. In addition, long-term results suggest that tall oil-based rejuvenators may retain stiffness reduction effects better than some alternatives; however, full recovery below the original RA binder stiffness may not be maintained after extended aging [94,101].

### 2.7. Animal-Based Rejuvenators

Animal-based bio-rejuvenators are a relatively limited but developing group. Most available studies focus on swine manure-derived bio-binders and swine manure/algal hybrid systems. Other animal-origin materials, including waste pig fat, chicken fat oil, tallow, poultry fat oil, fish oil waste, butter waste, and meat-processing oils, have also been proposed as potential rejuvenator sources. Their fat content, long-chain fatty acids, amide-rich compounds, and light molecular fractions may soften aged bitumen by replenishing maltenes, reducing viscosity, restoring ductility, and improving low-temperature performance. Swine manure-derived bio-binders have been widely examined for aged asphalt, RAP, and RAS systems. In RAS-modified binders, the use of 10% swine manure bio-binder reduced viscosity and compensated for the stiffening effect of RAS up to 30% RAS content; it also lowered the limit cracking temperature by 4–5 °C and increased ductility by 160–259%, depending on RAS content [108]. In high-RAP mixtures, the incorporation of 5% bio-binder improved the cracking resistance of 40% RAP mixtures, increasing overlay test cycles from 37 to 523 while maintaining acceptable rutting and moisture resistance [109]. At the binder scale, 10% swine manure bio-binder reduced the stiffness of PAV-aged asphalt by 63%, increased the m-value by 23%, and improved fracture energy to 132% of the 2PAV-aged binder, indicating enhanced relaxation and cracking resistance [110]. At the molecular level, these improvements were linked to reduced large molecular size fractions, improved colloidal stability, and weakened intermolecular interactions; for instance, 10% bio-binder reduced the colloidal instability index from 0.61 to 0.51, while hexadecanamide increased asphaltene stacking distance by 2.5 Å and vertical separation by 5 Å [110,111]. These effects were mainly associated with the interaction of amide-rich molecules with asphaltene structures, which altered conformational packing and promoted the partial deagglomeration of aged asphaltene clusters [110,111,112].

Further studies have focused on hybrid swine manure/algal bio-rejuvenators, which combine the lipid-rich character of swine manure with the protein- and nitrogen-rich composition of algae. These hybrid systems showed better rejuvenation performance than swine manure- or algal-derived bio-oils used alone. In particular, the 4:1 algal/swine manure system reduced the asphaltene content of aged asphalt by 21% and gave the highest colloidal stability improvement among the tested ratios [112,113]. This improvement was attributed to the ability of algal-derived nitrogen-containing molecules to open oxidized asphaltene stacks, while swine manure-derived long-chain molecules intercalated into the enlarged spacing and facilitated deagglomeration [112,113,114]. Quantitatively, the co-liquefied hybrid rejuvenator produced higher increases in the crossover modulus and crossover frequency than single-source systems, with crossover modulus values of 2.50 × 10^6^ Pa for the hybrid system compared with 1.99 × 10^6^ Pa for manure-based and 2.13 × 10^6^ Pa for algae-based rejuvenators and crossover frequency values of 3.39 Hz, 0.31 Hz, and 1.56 Hz, respectively [113,114]. Nevertheless, high rejuvenation capacity does not necessarily indicate long-term durability. Some animal-based bio-rejuvenators can effectively restore rheological properties, but their resistance to moisture damage and secondary aging may differ depending on molecular composition and polarizability. Lower-polarizability systems, such as Rej-Swilgae, showed better resistance to moisture and aging than some single-source rejuvenators. From a molecular perspective, lower-polarizability molecules are less easily distorted under oxidative conditions and therefore have a lower tendency to form reactive polar intermediates, free radical species, and oxygen-containing functional groups. This can slow oxidative chain reactions, limit secondary hardening, and help preserve the restored colloidal balance of the aged binder. Therefore, animal-based rejuvenators should be selected not only according to their immediate softening and rheological recovery effects but also considering their oxidation susceptibility, moisture resistance, and long-term durability after rejuvenation [115,116].

In addition to swine manure-based systems, waste animal fats have also been evaluated as direct animal-based rejuvenators. Waste pig fat was reported to restore the maltene/asphaltene balance of RAP binder, improve compatibility with aged asphalt, reduce viscosity and mixing compaction temperatures, and enhance low-temperature and fatigue cracking resistance [117]. Similarly, waste chicken fat oil (CFO) was used as a biomass rejuvenator for laboratory-aged asphalt. In that study, 6% CFO was suggested as an effective dosage. CFO softened aged asphalt, improved fatigue properties, delayed fatigue damage, reduced the sulfoxide index, and enhanced low-temperature stress relaxation. However, CFO mainly acted through physical blending rather than a chemical reaction, and excessive dosage could reduce rejuvenation efficiency [118]. Tallow or refined tallow has also been considered in broader rejuvenator comparison studies on high-RAP mixtures. These results indicate that animal fat-derived products may contribute to aged binder softening and low-temperature performance recovery, although binder-level mechanistic evidence for tallow is still more limited than that for swine manure or waste chicken fat oil [119]. Overall, the animal-based group remains less mature than plant oil, biomass oil, or tall oil systems, but the available quantitative evidence indicates a usable preliminary dosage window. Waste pig fat has been tested mainly at 3–9 wt.%, swine manure bio-binder has been evaluated in RAS and 40% RAP systems, hybrid swine manure/algal rejuvenators have been assessed using molecular and rheological recovery indices, and CFO has an indicated optimum near 6%. Future studies should report fatty acid composition, nitrogen- or protein-derived functional groups, moisture content, ash or mineral residue, volatility, odor and emission behavior, and storage stability so that these materials can be graded before asphalt application.

### 2.8. Other Biowastes

Other agricultural by-products and biowastes have also been considered potential bio-rejuvenators for recycled asphalt binders. Hu et al. [120] produced two bio-rejuvenators from municipal wastes composed of mixed waste plastics, crude sewage sludge, and agricultural waste through pyrolysis. These materials reduced stiffness and temperature sensitivity and improved fatigue performance, although full rheological recovery was not obtained. Similarly, Jalkh et al. [121] suggested that oil extracted from spent coffee grounds could be used for recycling aged bitumen. Ren et al. [122] further showed that the combined use of bio-oil and lignin produced a synergistic effect. In this system, bio-oil improved flexibility and fatigue resistance, whereas lignin contributed to structural stability, high-temperature performance, and aging resistance.

Food waste- and algal-derived oils have also been examined within this non-conventional group. Abdalla et al. [123] reported that food waste bio-oil improved the rheological response and low-temperature relaxation capacity of aged and RAP binders while reducing the carbonyl index and aging rate. Tabaković et al. [124] evaluated microalgal oil obtained from Nannochloropsis oceanica biomass and indicated its possible use as a rejuvenator for aged bitumen. However, both food waste bio-oil and microalgal oil still need further evaluation, particularly in terms of binder compatibility, dosage sensitivity, and long-term performance.

Sludge-derived oils represent another waste-based subgroup that has been investigated for asphalt recycling. Lee and Minh Le [125] used sewage sludge bio-oil as a rejuvenator in RAP mixtures and reported improvements in flexibility, fatigue life, tensile strength, and durability. Li et al. [126] prepared bio-asphalt using municipal sludge-derived heavy oil and found good compatibility with petroleum asphalt, improved low-temperature performance, and acceptable storage stability at suitable dosages. However, excessive oil content reduced high-temperature performance. In general, this group includes municipal waste-derived pyrolysis products, spent coffee ground oil, food waste bio-oil, microalgal oil, lignin/bio-oil composites, sewage sludge bio-oil, and sludge-derived heavy oil. Their main effects are related to waste valorization, stiffness reduction, improved fatigue or low-temperature performance, and partial rheological recovery.

Because this section includes municipal, food, algal, coffee ground, and sewage sludge sources, these materials are better interpreted as other biogenic or organic waste-derived rejuvenators rather than strictly agricultural by-products. Their quantitative evidence is highly dispersed: optimum or tested dosages range from 2 to 4% sewage sludge bio-oil in mixture studies to 5% food waste bio-oil, 5–10% sludge-derived heavy oil, 10 wt.% bio-oil/lignin systems, and 14% municipal waste-derived rejuvenators. Their common advantage is waste valorization combined with stiffness reduction and cracking performance recovery, but their chemistry is more heterogeneous than conventional vegetable oil or tall oil products. Practical use therefore requires the tighter control of contaminants, water and ash content, pyrolysis or extraction conditions, heavy metal risk in sludge-derived oils, odor and emission behavior, and post-aging performance.

## 3. Discussion

The reviewed literature indicates that bio-rejuvenation is a multi-path recovery process rather than a simple viscosity reduction treatment. Agricultural by-products, biowastes, and other biogenic materials can reduce binder stiffness and improve cracking-related performance, but the extent and durability of this recovery depend on feedstock chemistry, processing route, dosage, aged binder condition and diffusion ability. For this reason, the reviewed materials are most usefully compared through a unified framework that links source, functional chemistry, dominant mechanism, performance benefit, and implementation risk.

Mechanistically, three overlapping but non-equivalent pathways can be distinguished. First, physical softening occurs when low-viscosity oils diffuse into aged bitumen and replenish light fractions, thereby increasing molecular mobility and reducing viscosity or the complex modulus. Second, colloidal and interfacial rejuvenation occurs when polar, aromatic, phenolic, amide-rich, or resinous compounds weaken oxidized asphaltene aggregation, improve maltene–asphaltene balance, or reduce aging-related carbonyl and sulfoxide indices. Third, reactive modification occurs when functionalized oils, such as epoxidized, acrylated, or hybrid systems, participate in chemical or polymer network interactions that may improve compatibility, elastic recovery, or aging resistance. These pathways explain why similar penetration or viscosity recovery can lead to different long-term and mixture-scale outcomes.

Table 4 was constructed to provide a quantitative synthesis of the rejuvenation efficiency of different bio-rejuvenator classes relative to aged binders. For this purpose, the most frequently reported physical properties, including penetration, softening point, and rotational viscosity, together with key rheological parameters such as rutting factor (G*/sinδ), fatigue factor (G*sinδ), and, where available, low-temperature stiffness-related indicators, were selected as comparison criteria. Studies reporting these parameters were included as key sources for each bio-rejuvenator category. The optimum dosage values were extracted from the corresponding studies based on the dosage levels at which the most balanced restoration of physical and rheological properties was achieved. For each performance parameter, the minimum and maximum improvement or reduction ranges were determined relative to the aged binder. When numerical values were not directly tabulated in the original studies, approximate values were carefully estimated from the reported figures and graphs, provided that the graphical data were sufficiently clear and measurable. Parameters that were not investigated or not explicitly reported in the reviewed studies were indicated as NR (not reported), thereby avoiding unsupported assumptions and preserving the transparency of the comparative synthesis.

The quantitative comparison in Table 4 shows that bio-rejuvenator classes differ markedly in their dominant restoration mechanisms. Waste cooking oils exhibit the strongest penetration recovery, reaching up to 790%, together with substantial reductions in G*/sinδ of up to 90% and G*sinδ of 65–80%, indicating a powerful softening and fatigue recovery effect but also a potential risk of excessive softening. Agricultural and forestry residue-derived rejuvenators also show a strong conventional recovery profile, with penetration increases up to 598%, viscosity reductions of 76–81%, and G*/sinδ decreases of 66–82%, suggesting high efficiency in restoring workability and rutting-related rheological balance. Biomass-derived bio-oils provide a relatively broad but more moderate response, with penetration recovery up to 432% and G*/sinδ reductions ranging from 12% to 75%, reflecting their source- and process-dependent performance. Reactive rejuvenators show lower penetration recovery than waste oils and agricultural residues, with a maximum increase of 232%, but their G*/sinδ reduction of 65–85% indicates a more targeted rheological restoration mechanism. In contrast, the “others” category shows a limited penetration recovery of only 14–49% yet the highest rheological softening effect, with viscosity, G*/sinδ, and G*sinδ reductions reaching 92%, 94%, and 90%, respectively. Overall, the data suggest that waste cooking oils and agricultural/forestry residues are more effective in restoring conventional physical properties, whereas reactive systems and some alternative bio-rejuvenators provide stronger rheological recovery. Therefore, bio-rejuvenator selection should be based on a balance between physical restoration, rheological improvement, and the risk of over-softening rather than on a single performance indicator.

Another important distinction concerns binder-level, mixture-level, and field-scale performance. Binder-level recovery is necessary to verify improvements in workability, viscoelastic balance, and cracking resistance; however, it does not necessarily guarantee mixture durability or long-term field performance. Mixture behavior is also influenced by RAP/RAS blending efficiency, aggregate structure, air void distribution, rejuvenator diffusion, moisture conditioning, freeze–thaw exposure, compaction quality, traffic loading, and climatic conditions. Moreover, laboratory aging and performance tests cannot fully reproduce the combined environmental and mechanical effects experienced during pavement service. Therefore, promising bio-rejuvenators should be validated through mixture-scale rutting, cracking, fatigue, moisture, and durability tests, followed by the long-term monitoring of field sections under different traffic and climatic conditions.

Long-term behavior remains one of the main barriers to implementation. Secondary aging can evaporate or oxidize light components, reduce the initial softening benefit, and re-establish asphaltene-rich structures. Compatibility problems, phase separation, storage instability, moisture-driven migration, and the preferential adsorption of rejuvenator molecules onto aggregate surfaces may further reduce durability. These risks are the most critical for low-viscosity oils, highly oxygenated pyrolysis products, sludge-derived oils, and poorly characterized waste streams. Pretreatment, esterification, fractionation, blending with stabilizers, encapsulation, or hybrid formulation can mitigate some of these problems, but these solutions must be evaluated against cost, emissions, and construction practicality.

The sustainability argument also requires more quantitative evidence. Many bio-rejuvenators reduce virgin binder demand, support RAP/RAS reuse, and valorize waste streams, but the net environmental benefit depends on collection distance, drying, extraction or pyrolysis energy, chemical pretreatment, additive dosage, storage, plant temperature, and service life extension. For this reason, future evaluations should couple binder and mixture performance with life-cycle assessment (LCA), techno-economic analysis, and sensitivity analysis. Without consistent system boundaries and performance-based service life assumptions, a material that appears sustainable at the feedstock stage may not necessarily provide the lowest carbon footprint at pavement scale.

Feedstock variability must be controlled because it directly affects rejuvenator chemistry and performance. For oil-based products, fatty acid profile, free fatty acid content, acid value, moisture, and oxidation level are key parameters. For biomass-, sludge-, algal-, and manure-derived products, ash content, mineral residue, protein/lipid ratio, oxygenated compounds, and nitrogen-containing groups are also important. Practical implementation therefore requires raw material grading, moisture reduction, filtration or de-ashing, acid value adjustment, compositional screening, and batch-to-batch quality control.

Based on the reviewed evidence, the most urgent research needs are: the standardized reporting of feedstock chemistry and aging protocols; comparable dosage selection methods; integrated binder–mixture–field validation; long-term aging and moisture conditioning protocols; hybrid or reactive formulations that balance cracking and rutting; and LCA-supported implementation guidance. This roadmap is particularly important for animal-based and other non-conventional waste-derived rejuvenators, where promising mechanisms have been reported but the evidence base remains narrower and less standardized than for WCO, VVO, biomass-derived, or tall oil-based systems.

## 4. Conclusions

This review shows that agricultural by-products, biowastes, and other biogenic materials can contribute to aged bitumen rejuvenation through several complementary mechanisms. The most important conclusion is that bio-rejuvenation should not be interpreted as a single softening phenomenon. Depending on the feedstock and processing route, recovery may involve light fraction replenishment, colloidal rebalancing, asphaltene deagglomeration, chemical or interfacial interaction, polymer network repair, anti-aging effects, or combinations of these processes.

The main conclusions drawn from the revised review are as follows:

- WWOs and VVOs are widely available and effective in restoring flexibility and reducing stiffness. However, excessive dosage may impair high-temperature performance and rutting resistance.
- Reactive and chemically modified bio-oils can provide a more balanced recovery by combining softening with improved compatibility, rheological response, and aging resistance.
- Biomass-derived oils, agricultural and forestry residues, and tree resin-derived products show strong potential, but their performance is highly dependent on feedstock composition, processing method, and dosage.
- Animal-based rejuvenators and other biogenic waste streams broaden the resource base, but their evidence base is more limited and heterogeneous. Swine manure/algal hybrids and waste chicken fat oil provide promising mechanistic evidence, whereas tallow, fish oil waste, butter waste, sewage sludge oil, spent coffee ground oil, algal oil, and food waste oils still require broader validation.
- Binder-level recovery alone is insufficient to confirm long-term pavement performance. Mixture-scale testing and field validation are required to assess rutting, cracking, moisture damage, fatigue, and durability.
- The main unresolved issues include dosage optimization, feedstock variability, compatibility, volatility, moisture susceptibility, secondary aging, storage stability, and the lack of standardized evaluation procedures.
- Future research should integrate feedstock grading, chemical fingerprinting, balanced mix design, long-term aging protocols, field sections, LCA, and techno-economic analysis so that bio-rejuvenator selection is based on both engineering reliability and verified environmental benefit.

In summary, bio-rejuvenators can become an important material strategy for recycled asphalt pavements when they are selected and dosed through performance-based criteria. Their strongest contribution lies in enabling the higher use of reclaimed asphalt materials while reducing dependence on virgin binder and creating value from waste streams. However, successful implementation requires moving from material availability toward verified performance reliability, especially under long-term aging, moisture exposure, and mixture-scale service conditions.

## Figures and Tables

**Table 1:** Summary of WCO-based rejuvenators for aged bituminous binders.

References	Usage/Process	Improvements	Limitations
[24,27]	WCO direct-blended with aged binder	penetration recovery viscosity reduction improved low-temp flexibility	excessive softening risk dosage-sensitive response
[25,26]	WCO and WEO directly added to RAP-extracted aged binder	lower dosage than WEO enhanced asphaltene/maltene balance decline in carbonyl/sulfoxide intensity	high-temperature stiffness reduction optimum dosage required
[28]	WCO directly incorporated into base binder	improved stress relaxation lower creep stiffness good storage compatibility	reduced deformation resistance lower elastic recovery
[29,30]	WCO with encapsulation and delayed release into aged bitumen	controlled rejuvenator release viscosity/penetration recovery anti-aging functionality	microcapsule design complexity field validation needed
[31]	microencapsulated waste soybean cooking oil (WSCO)	improved thermal stability controlled WSCO permeation	temp-sensitive permeation complex preparation route
[32]	directly blending WSCO with aged binder	reduce viscosity and stiffness enhanced fatigue performance	limited low-temp performance climate-dependent optimum dosage selection
[33,34]	WCO used with transesterification or chemical pretreatment	improved rutting resistance reduced moisture susceptibility better rheological stability	additional processing step quality control required
[35,36]	direct blending with RAP binder	improved fatigue resistance restored viscoelastic response enabled higher recycled binder content	high WCO increases viscous strain rutting parameter may decrease
[37]	fraction-based rejuvenation and secondary aging evaluation	improved aging resistance lower volatility than virgin light fractions better long-term stability for selected fractions	performance depends on carbon chain length fraction selection is critical
[38]	blended WCO sources under secondary aging	improved fatigue life practical WCO source consideration	source variability affects efficiency mono-source is impractical
[39]	WCO combined with plasticizer, toughener, and petroleum resin	reduced C=O and S=O groups improved viscoelastic behavior molecular-level rejuvenation insight	composite system masks WCO-only effect UV aging-specific scope
[40]	directly blending mixed WCO and WEO	improved workability enhanced fatigue behavior	field validation needed optimum dosage required
[41]	WCO used after catalytic esterification	improved fatigue resistance better high/low-temperature balance	chemical treatment increases complexity
[42]	WCO used with LDPE in full RAP recycling	improved recycling feasibility polymer compensated softening effect	binder-specific mechanism limited

**Table 2:** Summary of biomass-derived bio-oils used as rejuvenators.

Reference	Biomass Source	Dosage (wt%)	Aging Condition	Process
[70]	waste wood	10	RTFO- and PAV-aged	thermochemical liquefaction
[71]	waste wood	10, 15, 20	RTFO- and PAV-aged	fast pyrolysis
[72]	sawdust	10, 15, 20	RTFO- and PAV-aged	fast pyrolysis
[73]	corncob and birch bark	5, 10	RTFO- and PAV-aged	fast pyrolysis
[74]	plant-based bio-oil	15, 30	high-RAP mixture	fast pyrolysis
[75]	waste sawdust	5, 10, 15, 20	TFOT-aged	thermochemical liquefaction
[76]	harvested crop	1, 1.5, 2	50% unaged and 50% RAP mixture	commercial bio-oil direct blending
[77]	straw	3, 6, 9, 12	RTFO- and PAV-aged	fast pyrolysis
[78]	biodiesel by-product	1, 2, 3	unaged, RTFO- and PAV-aged	direct blending
[79]	biodiesel waste	10	high-RAP mixture	direct blending
[80]	corncob, birch bark, sawdust, pine bark, peanut shells	10	secondary-aged (TFOT + PAV)	thermochemical liquefaction
[81]	sugarcane bagasse and rice straw	5, 10, 15, 20	RTFO- and PAV-aged	fast pyrolysis
[82]	waste wood	2, 4, 6, 8, 10	RTFO- and PAV-aged	fast pyrolysis
[83]	rice husk	0.5–10	RTFO-, PAV- and UV-aged	fast pyrolysis

Abbreviations: RTFO, rolling thin film oven; PAV, pressure aging vessel; TFOT, thin film oven test; UV, ultraviolet.

**Table 3:** Tree resin-derived bio-rejuvenators and their restoration effects.

Reference	Source	Product	Aging Condition	Key Findings
[94]	tall oil	tall oil fatty acids; distilled tall oil; rosin	RTFO- and PAV-aged	enhanced chemical stability recovered low-temp performance and fatigue life
[96]	tall oil	tall oil-based rejuvenator	RTFO- and PAV-aged	reduced asphaltene aggregation improved microstructure
[97]	tall oil	tall oil-derived phytosterol and fatty acid-based oil	30% RAP binder	improved cracking resistance no adverse rutting effect
[98]	tall oil	tall oil fatty acid-based rejuvenator	50% RAP binder	restored RAP binder performance reduced global warming potential and cost
[99]	tall oil	tall oil-based rejuvenator	20%, 50% and 70% RAP binder	improved cracking resistance balanced rutting–cracking response
[100]	tall oil	tall oil-based rejuvenator	70% RAP binder	improved low-temp cracking resistance maintained rutting resistance
[101]	tall oil	tall oil-based rejuvenator	100% RAP binder	highest long-term stiffness reduction acceptable rutting performance
[102]	tall oil	tall oil-based rejuvenator	25% and 45% RAP binder	lowered true PG improved fatigue resistance
[44]	tall oil	distilled tall oil-based rejuvenator	100% RAP binder	reduced RAP binder grade improved fatigue performance
[103]	rosins, esters, fatty acids	Bitutech RAP/Hydrogreen (commercial)	15% and 50% RAP binder	improved moisture resistance softened RAP binder
[104]	plant extracts/rosin-based source	Hydrogreen (commercial)	40% RAP and 25% RAP + 5% RAS binder	comparable field performance supported high RAP/RAS use
[95]	pine oil	Sylvaroad RP1000 (commercial)	100% RAP binder; RTFO-aged after rejuvenation	recovered PG improved fatigue tolerance
[105]	tall oil and rosin	Sylvaroad RP1000 (commercial)	25%, 50%, and 70% RAP binder	reduced binder viscosity improved fatigue resistance
[96]	tall oil	tall oil-based rejuvenator	RTFO- and PAV-aged	reduced elastic modulus resisted secondary aging
[106]	tall oil and mixed bio-sources	tall oil-based rejuvenator	RTFO- and PAV-aged	improved fatigue response mainly physical softening
[107]	rosin	rosin-based catalytic rejuvenator	TFOT- and PAV-aged	improved low-temp resistance better fatigue performance

**Table 4:** Quantitative comparison of optimum bio-rejuvenator effects relative to aged binder.

Bio-Rejuvenator Type	Key Sources	Optimum Dosage (%)	Penetration Increment (%)	Softening Point Decrease (%)	Viscosity Decrease (%)	G*/sin δ Decrease (%)	G*sin δ Decrease (%)	Creep Stiffness (S) Decrease (%)
Waste cooking oils	[26,29,31,32,34,35,37,41]	3.5, 5, 5.2, 8, 10, 12.5	80–790	13–35	28–75	50–90	65–80	20–89
Virgin vegetable oils	[43,46,47,53,56]	3.4, 6, 7, 9, 10	14–450	8–38	57–75	43–76	26–76	46–54
Reactive rejuvenators	[60,61,66,68,69]	6, 7, 10, 13	16–232	8–23	49–58	65–85	*NR	31–80
Biomass- derived bio-oils	[71,72,73,75,76,78,81,82,83]	3, 8, 10, 15, 20	85–432	12–25	35–59	12–75	29–43	37–59
Agricultural and forestry residues	[85,86,87,88,89,90]	5, 7, 10, 12.4	52–598	7–30	76–81	66–82	NR	58–82
Tree resin- derived	[94,95,96,107]	1.5, 8, 9	17–184	6–16	44–82	25–83	NR	11–44
Animal-based rejuvenators	[108,110,115,117,118]	3, 9, 10, 15	75–390	7–16	52–75	33–87	42–76	4–82
Others	[120,122,126]	10, 18, 20	14–49	4–8	67–92	74–94	84–90	48–57

*NR: Not reported.

## Data Availability

No new data were created or analyzed in this study. Data sharing is not applicable to this article.
